# Trends in non-pharmacological treatment for insomnia: A nationwide study

**DOI:** 10.1371/journal.pone.0334142

**Published:** 2025-11-07

**Authors:** Mingee Choi, Junbok Lee, Jeahyung Lee, Suonaa Lee, Eun Lee, Jaeyong Shin

**Affiliations:** 1 Department of Preventive Medicine, Yonsei University College of Medicine, Seoul, Korea; 2 Institute for Innovation in Digital Healthcare, Yonsei University, Seoul, Korea; 3 Department of Economics, College of Commerce and Economics, Yonsei University, Seoul, Korea; 4 Department of Psychiatry and the Institute of Behavioral Science in Medicine, Yonsei University College of Medicine, Seoul, Republic of Korea; Ajou University School of Medicine and Graduate School of Medicine, KOREA, REPUBLIC OF

## Abstract

**Background:**

As the prevalence of insomnia disorder in Korea is gradually increasing, understanding real-world treatment patterns is essential for designing appropriate clinical guidelines. Although non-pharmacological treatments are recommended as first-line interventions, their actual use in clinical settings remains insufficiently studied. This study examined the current status of non-pharmacological treatments for insomnia implemented by Korean doctors.

**Methods:**

Data from the National Health Insurance Service (NHIS) sample cohort, comprising 518,119 patients diagnosed with insomnia disorder (ICD-10 code: G47) between 2002 and 2019, were analyzed. Treatment types at the time of first diagnosis and the timing of non-pharmacological treatment initiation were assessed.

**Results:**

Only 0.01% of patients received non-pharmacological treatment at their first diagnosis, and 97.5% did not receive such treatment during the study period. Among those who eventually received it, the interval from diagnosis to treatment initiation decreased over time. A modest increase in the use of non-pharmacological treatments was observed after 2018, following changes in national insurance coverage.

**Conclusion:**

The findings highlight a significant gap between clinical guidelines and real-world practice. Although non-pharmacological treatments remain underutilized, recent improvements suggest potential for better integration into routine care, emphasizing the need for ongoing efforts to enhance access to recommended therapies.

## Introduction

Insomnia disorder, which refers to the symptoms of not getting normal sleep, is a common disease with an increasing prevalence worldwide [1–6]. Transient episodes of short-term insomnia can occur extensively, resulting in chronic insomnia, which currently affects 30–50% of the adult population [7]. Therefore, it is necessary to manage the disease burden through appropriate nationwide management.

Treatment guidelines in several countries, including the United States, Europe, and Korea, recommend non-pharmacological treatment for insomnia, such as cognitive behavioral therapy, as the primary [8–14], as various clinical studies have shown their effectiveness in improving insomnia [15]. However, it is not practiced because it is time-consuming and expensive, and there is a lack of experts who can provide this treatment [16].

For individuals whose symptoms are difficult to treat with non-pharmacological treatments or whose condition does not improve after the treatment, prescribing hypnotics may be considered. However, hypnotics carry the risk of abuse, dependence, falls, and delirium. In Korea, concerns have been raised regarding the excessive use of sleeping pills [17], benzodiazepines, antipsychotics, and antidepressants [18].

This study aimed to confirm the initial treatments offered to patients diagnosed with insomnia disorder, specifically focusing on the proportion of patients who received non-pharmacological treatments. As the prevalence of insomnia disorder in Korea is gradually increasing [19], it is important to understand the treatment status to lay the foundation for designing clinical guidelines for patients with insomnia disorder. Therefore, we investigated the non-pharmacological treatment status in patients with insomnia disorder (ICD-CODE: G47) between 2002 and 2019 using data from the National Health Insurance Service (NHIS).

## Methods

### Study design

We examined the initial treatment provided to patients diagnosed with insomnia disorder, the time taken to provide non-pharmacological treatment, and detailed treatment status. The NHIS sample cohort data treat codes for psychiatric illnesses as sensitive information and do not provide them to researchers. Therefore, information on F51, a psychiatric insomnia code, could not be obtained, and the study was conducted using only the information on G47. This study used secondary data in which all personal information was anonymized and de-identified. The requirement for ethical approval was waived by the Institutional Review Board of the Yonsei University Health System (4-2023-0475). Data from the NHIS sample cohort were accessed and analyzed for research purposes from June 10, 2023, to May 31, 2024.

### Data and study population

This study analyzed NHIS sample data. The sample data are 2% of the national population eligible for health insurance and medical benefits, stratified by sex, age, type of insurance, and region. The data comprised demographic characteristics (type of insurance, age, and sex) and medical use status (diagnosis and treatment). This study attempted to analyze treatment behavior by counting prescription details at the first diagnosis and the non-pharmacological treatments, specifically including personal mental therapy, group mental therapy, family therapy, cognitive behavior therapy, and electroshock therapy.

The analysis was conducted on 518,119 patients diagnosed with insomnia disorder (ICD-10 code: G47) between 2002 and 2019 [20]. We analyzed patients who were initially diagnosed with insomnia disorder and tracked their non-pharmacological treatment status from the initial diagnosis to December 31, 2019, for the analysis.

### Statistical analysis

All analyses were conducted using SAS software (version 9.4; Cary, NC, USA). Continuous variables were summarized as mean and standard deviation (SD), and categorical variables were presented as number and percentage (N, %). In addition, chi-square tests were used to compare demographic characteristics across patient groups according to the timing of non-pharmacological treatment.

## Results

### Trend in patients with insomnia disorder

**Fig 1:**
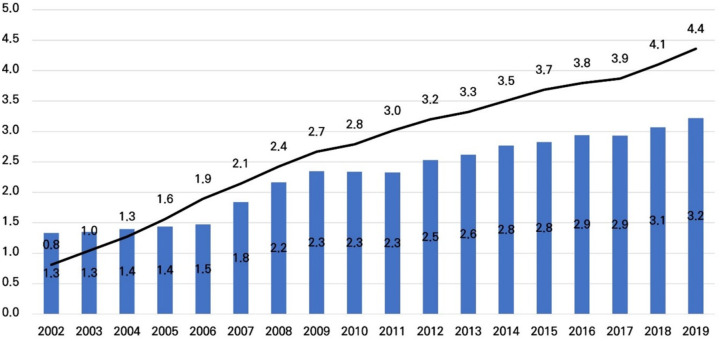
Annual incidence of insomnia disorder (ICD-CODE: G47) Bar: rate of insomnia disorder relative to all patients; Line: average number of outpatient visits for patients with insomnia disorder.

The number of people diagnosed with insomnia disorder per year continued to increase during the observation period from 7,937 in 2002–46,451 in 2019 (Table 1). The ratio of insomnia disorder to all patients increased from 1.3% in 2002 to 3.2% in 2019, and the average number of outpatient visits by patients with insomnia disorder increased over time (Fig 1).

**Table 1:** General information of patient who diagnosis insomnia disorder (N = 518,119).

Year	Total	Age		Women	Type of Insurance			Income				
					Local-subscriber	Employed	Medical aid	1(row)	2	3	4	5(high)
	N	mean	sd	%	%	%	%	%	%	%	%	%
**2002**	7937	73.0	16.3	69.0	49.5	50.3	0.3	14.3	14.5	20.6	22.8	27.9
**2003**	10288	72.8	16.1	67.5	48.3	51.6	0.2	15.1	14.0	17.9	23.7	29.3
**2004**	12745	72.4	163	66.9	47.3	52.5	0.2	14.8	15.2	18.0	22.7	29.4
**2005**	15794	72.5	16.2	66.6	44.5	52.3	3.3	16.7	13.1	18.2	21.3	30.7
**2006**	19313	72.7	16.2	65.4	38.1	51.5	10.5	16.5	14.9	15.0	23.1	30.5
**2007**	22126	72.5	16.4	65.3	36.9	52.6	10.5	15.8	13.6	17.4	22.3	31.1
**2008**	25092	72.0	16.5	65.3	35.6	54.0	10.4	15.0	14.4	17.0	22.2	31.4
**2009**	27696	71.5	16.4	65.0	34.2	55.2	10.6	15.5	14.3	17.2	21.8	31.1
**2010**	29070	71.0	16.5	64.8	34.1	56.2	9.7	15.5	14.6	16.9	21.9	31.2
**2011**	31533	70.5	16.5	63.6	33.5	57.0	9.5	16.1	14.0	16.9	21.6	31.5
**2012**	33596	70.0	16.4	63.4	32.3	58.7	9.1	15.6	14.8	16.3	21.7	31.6
**2013**	35003	69.4	16.4	63.1	31.8	59.6	8.6	16.1	14.4	16.5	21.5	31.6
**2014**	37048	68.5	16.5	63.0	30.7	61.1	8.1	16.3	14.1	16.7	20.8	32.1
**2015**	39110	68.0	16.5	62.4	30.2	62.1	7.7	16.7	14.0	16.4	21.0	31.9
**2016**	40382	67.0	16.5	62.4	29.5	62.4	8.1	23.0	13.4	14.9	19.6	29.1
**2017**	41219	66.7	16.5	62.0	29.0	62.8	8.2	23.7	12.8	14.6	19.2	29.8
**2018**	43716	65.7	16.6	62.0	29.3	62.7	8.0	24.1	12.7	14.5	19.3	29.5
**2019**	46451	64.9	16.7	60.9	29.6	62.6	7.8	24.9	11.5	14.9	19.1	29.7
**Total**	518119	69.1	20.1	63.5	33.2	58.7	8.1	18.6	13.7	16.2	21.0	30.7

Annual counts may include the same patient across years; the total N (518,119) represents unique patients from 2002–2019.

### Treatment at first diagnosis of insomnia disorder

Table 2 shows the distribution of treatment modalities received at the time of first diagnosis of insomnia disorder. Percentages represent the relative proportion of each treatment type among all treatments provided at the first diagnosis. Non-pharmacological treatments were prescribed to approximately 0.1% of the patients. The proportion of prescribed medications at the first diagnosis tended to decrease, whereas the proportion of counseling initially tended to increase. Anesthesia and imaging were excluded from the interpretation as they were not considered as a treatment for insomnia disorder and seemed to be because of other complications.

**Table 2:** Treatment at first diagnosis of insomnia disorder (%).

Year	Consultation	Medication	Injection	Physical therapy	Non-pharmacological therapy	Procedure and surgery	Examination	others
**2002**	90.96	3.29	4.47	0.48	0.00	0.02	0.67	0.11
**2003**	94.29	2.42	2.24	0.27	0.00	0.03	0.64	0.11
**2004**	96.27	1.46	1.41	0.21	0.01	0.02	0.56	0.06
**2005**	97.08	0.84	1.44	0.20	0.00	0.02	0.37	0.05
**2006**	97.37	1.05	1.04	0.19	0.03	0.02	0.24	0.06
**2007**	98.02	0.66	0.75	0.13	0.03	0.11	0.26	0.04
**2008**	98.42	0.55	0.52	0.15	0.02	0.05	0.25	0.04
**2009**	98.71	0.25	0.43	0.15	0.00	0.21	0.20	0.05
**2010**	98.40	0.25	0.42	0.14	0.01	0.46	0.27	0.05
**2011**	98.41	0.22	0.48	0.13	0.00	0.23	0.31	0.22
**2012**	98.38	0.15	0.20	0.05	0.00	0.79	0.37	0.06
**2013**	98.27	0.35	0.18	0.03	0.01	0.76	0.37	0.03
**2014**	98.75	0.45	0.14	0.03	0.01	0.13	0.45	0.04
**2015**	99.16	0.21	0.07	0.03	0.01	0.01	0.45	0.06
**2016**	99.08	0.17	0.06	0.07	0.01	0.00	0.52	0.09
**2017**	99.14	0.23	0.03	0.04	0.01	0.00	0.53	0.02
**2018**	99.01	0.00	0.22	0.00	0.13	0.00	0.00	0.64
**2019**	99.15	0.00	0.13	0.00	0.06	0.01	0.00	0.65
**Total**	97.72	0.7	0.79	0.13	0.02	0.16	0.36	0.13

**Fig 2:**
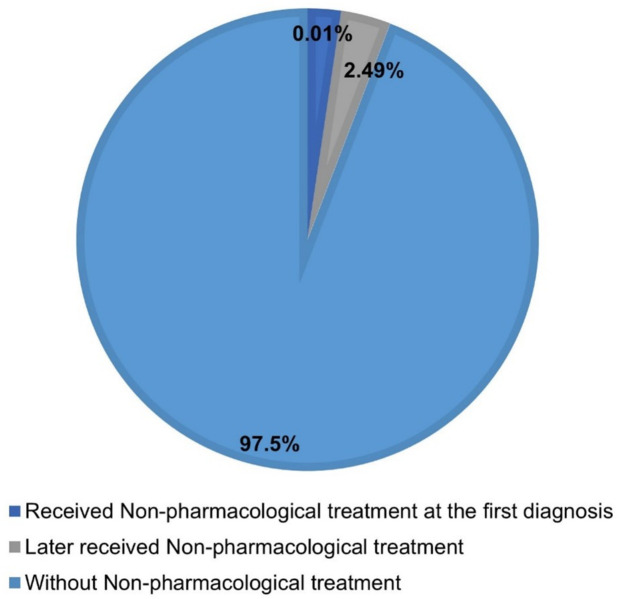
Distribution of non-pharmacological treatment related to insomnia disorder (2002–2019).

**Fig 3:**
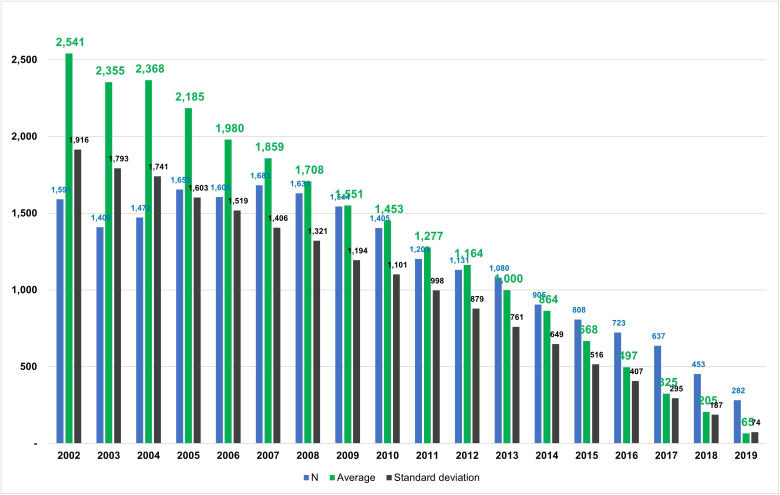
Mean interval from initial diagnosis to initiation of non-pharmacological treatment by year. The green bars indicate the mean interval from the initial diagnosis of insomnia disorder to the initiation of non-pharmacological treatment. The blue bars represent the year-specific number of patients who initiated non-pharmacological treatment after an initial diagnosis without it. The black bars denote the standard deviation (SD).

Fig 2 shows the distribution of non-pharmacological treatments administered during the first diagnosis. Approximately 0.01% of patients received non-pharmacological treatment from the first diagnosis. Fig 3 presents the mean interval by year of initial diagnosis, demonstrating a decreasing trend over time.

Table 3 shows the detailed treatment status of patients who received non-pharmacological treatment at the first diagnosis of insomnia disorder. A total of 88 non-pharmacological treatments were reported, with individual supportive psychotherapy accounting for the largest proportion. Patients who did not receive non-pharmacological treatment at the first diagnosis but eventually received non-pharmacological treatment were most likely to receive individual supportive psychotherapy and often received short personal psychotherapy within 10 minutes (Table 4).

**Table 3:** Detailed treatment status of people who received non- pharmacological treatment at first diagnosis.

Code	NN001	NN002	NN003	NN011	NN012	NN013	NN031	NN061	Total
Year	Individual Psychotherapy I (~10 min)	Individual Psychotherapy Ⅱ (10 ~ 20 min)	Individual Psychotherapy Ⅲ (20 ~ 30 min)	Individual Supportive Psycho therapy	Individual Intensive Analytic Psycho therapy	Individual Intensive Psycho therapy	Family Therapy- Individual	Cognitive Behavioral Therapy-Individual	
**2004**	0	0	0	1	0	0	0	0	1
**2005**	0	0	0	1	0	0	0	0	1
**2006**	0	0	0	3	1	3	0	0	7
**2007**	0	0	0	9	0	3	0	0	12
**2008**	0	0	0	7	0	3	0	0	10
**2010**	0	0	0	1	0	5	0	0	6
**2011**	0	0	0	1	0	1	0	0	2
**2012**	0	0	0	0	1	2	0	0	3
**2013**	0	0	0	1	0	2	0	0	3
**2014**	0	0	0	3	1	1	0	0	5
**2015**	0	0	0	6	2	0	3	0	11
**2016**	0	0	0	8	0	0	0	0	8
**2017**	0	0	0	5	0	0	1	0	6
**2018**	2	0	0	1	0	0	1	0	4
**2019**	1	2	1	0	0	0	1	4	9
**Total**	3	2	1	47	5	20	6	4	88

**Table 4:** Detailed treatment status of patients who did not receive non-pharmacological treatment at first diagnosis but received it later.

Code	NN001	NN002	NN003	NN004	NN005	NN011	NN012	NN013	NN021	NN023	NN031	NN032	NN040	NN061	NN062	NN100	NN111	NN112	Total
Year	Individual Psychotherapy I (~10 min)	Individual Psychotherapy Ⅱ (10 ~ 20 min)	Individual Psychotherapy Ⅲ (20 ~ 30 min)	Individual Psychotherapy Ⅳ (30 ~ 40 min)	Individual Psychotherapy Ⅴ (40 min~)	Individual Supportive Psychotherapy	Individual Intensive Analytic Psychotherapy	Individual Intensive Psychotherapy	Supportive Expressive Group Psychotherapy	Psychodrama-Group	Family Therapy- Individual	Family Therapy-Group	Occupational or Recreation Therapy	Cognitive Behavioral Therapy-Individual	Cognitive Behavioral Therapy-Group	Psychiatric Emergency Treatment	Individual History Taking	Social Work Guidance	
**2002**	0	0	0	0	0	237	33	0	0	0	2	0	0	0	0	0	0	0	272
**2003**	0	0	0	0	0	418	48	0	0	0	9	0	0	0	0	1	0	0	476
**2004**	0	0	0	0	0	513	33	0	0	0	24	0	0	0	0	2	0	0	572
**2005**	0	0	0	0	0	665	29	30	0	0	18	0	0	0	0	0	1	0	743
**2006**	0	0	0	0	0	825	30	127	0	0	13	0	0	0	0	0	0	0	995
**2007**	0	0	0	0	0	743	38	159	0	0	24	0	0	0	0	0	0	1	965
**2008**	0	0	0	0	0	996	28	256	0	0	22	0	0	0	0	1	1	0	1304
**2009**	0	0	0	0	0	1374	46	225	0	0	39	0	0	0	0	0	0	0	1684
**2010**	0	0	0	0	0	1574	37	319	0	0	92	0	0	0	0	0	0	0	2022
**2011**	0	0	0	0	0	2045	33	367	0	0	114	0	3	0	0	0	0	0	2562
**2012**	0	0	0	0	0	2713	76	580	0	0	129	0	4	0	0	0	0	0	3502
**2013**	0	0	0	0	0	3229	70	696	0	0	203	0	1	0	0	2	0	1	4202
**2014**	0	0	0	0	0	3368	128	808	0	0	247	0	0	0	0	1	0	0	4552
**2015**	0	0	0	0	0	4185	180	877	0	0	382	0	0	0	0	1	0	0	5625
**2016**	0	0	0	0	0	4885	143	1444	2	0	432	0	0	0	0	0	1	0	6907
**2017**	0	0	0	0	0	5867	155	1715	0	2	477	0	4	0	0	5	1	0	8226
**2018**	3197	1288	255	78	49	3211	76	1017	2	0	572	0	5	4	0	6	0	0	9760
**2019**	7447	3150	604	224	148	0	0	0	0	0	655	1	21	8	6	2	0	0	12266
**Total**	10644	4438	859	302	197	36848	1183	8620	4	2	3454	1	38	12	6	21	4	2	66635

An additional chi-square analysis comparing patients who received non-pharmacological treatment at the first diagnosis, those who received it later, and those who never received it showed consistent demographic patterns: employed insurance holders, women, non-metropolitan residents, and higher-income patients accounted for the largest proportions in all groups (all p < 0.001) (S1 Table).

## Discussion

Our analysis showed that less than 0.1% of patients received non-pharmacological treatment at the initial visit.

We observed that the proportion of patients receiving non-pharmacological treatments for insomnia was relatively low, and most patients did not receive non-pharmacological treatments even at first diagnosis. Contrary to the recommendations of various clinical guidelines for insomnia disorder, it has been confirmed that non-pharmacological treatments are not properly used in Korea. This could be because of various reasons, such as compensation for non-pharmacological treatment, time, cost, and the medical environment. Except for consultations, drugs and injections accounted for the largest proportion of the first treatment, and this phenomenon is similar in many places worldwide. Drugs such as benzodiazepines are the most commonly used treatments in the United States and were used by approximately 6–10% of adults as of 2010 [21]. In the UK, there was a study on the increased use of sleeping pills by patients with insomnia [1].

While pharmacological treatment can be an effective and easy way to address insomnia disorder, it is not a cure-all and may be associated with dependence and abuse with long-term use. Various studies have shown that combining medication and non-medication treatments can effectively improve sleep; therefore, it is crucial to provide appropriate non-pharmacological treatments [22–25]. As awareness of non-pharmacological treatment improves among physicians and patients, non-pharmacological treatments will be offered earlier, as suggested by the guidelines. This improvement in therapeutic approach is expected to have a positive impact on the treatment of insomnia disorder in the future.

On a positive note, the number of patients who did not receive non-pharmacological treatment at the initial diagnosis but later received it continued to increase over time. This increase is clinically meaningful, as earlier initiation of non-pharmacological treatment can enhance long-term outcomes and reduce the limitations of pharmacological therapy alone. The Ministry of Health and Welfare of Korea included cognitive behavioral therapy in its National Health Insurance list when it reorganized the health insurance system to further revitalize non-pharmacological treatment in 2018. Media reports have stated that this has increased the number of hospitals providing psychiatric care, making it more accessible to patients.

Moreover, various services for patients with insomnia disorders have recently been developed to provide non-pharmacological treatment using digital technology. Several studies have been conducted to verify the effectiveness of digital healthcare technologies for improving insomnia disorder [26–28]. The advantage of digital technologies is that they can overcome time and space constraints, which means that patients can use them to receive medical care without visiting a hospital. In many countries, digital healthcare services related to insomnia disorder are institutionalized within the healthcare system, even to the point of being reimbursed. Digital healthcare-based non-pharmacological treatments for insomnia disorder are expected to increase access to care for these patients.

This study has several limitations. First, insomnia disorder was defined using only the ICD-10 code G47, because the NHIS sample cohort database classifies psychiatric diagnostic codes such as F51 as sensitive information and does not provide them to researchers. In 2013, 35,003 patients were identified using only the G47 code. However, a previous nationwide study that included both G47 and F51 codes reported 43,961 patients during the same period, leading to a discrepancy of 8,958 cases [29]. This difference likely reflects the masking of F-code diagnoses in the NHIS datasets due to their classification as sensitive conditions. Consequently, the findings may be subject to selection bias, and caution is warranted when generalizing these results to the broader insomnia population.

Second, these findings should be interpreted in the context of broader demographic dynamics. The observed increase in patient numbers may, at least in part, reflect the effects of population aging and shifts in population structure. In addition, this study did not examine demographic factors that may underlie the observed decrease in medication use and increase in counseling. Future research with expanded datasets, including psychiatric F-codes, will be needed to investigate these trends in greater depth. Furthermore, because the observation period ended in 2019, patients first diagnosed in 2019 may have initiated non-pharmacological treatment after 2020 and were therefore not captured in this study. This limitation may have introduced bias in estimating the mean interval for the most recent cohorts, potentially leading to either underestimation or overestimation.

Third, there was no information on non-payment drug treatments in the health insurance data; therefore, details could not be considered owing to the data limitation. Finally, although this study describes various mixtures measured from the data used, the possibility of residual mixtures cannot be completely ruled out. However, despite these limitations, this study is unique in that it is the first in Korea to investigate prescriptions at the time of first diagnosis in patients with insomnia disorder. The results of this study are particularly noteworthy because they present the basis for desirable policy proposals in the future through a practical analysis of the contents of the guidelines for insomnia disorder.

This study explored the treatment preferences of Korean doctors for patients with insomnia disorders. At the time of initial diagnosis of insomnia disorder, there were few non-pharmacological treatments. However, patients who did not receive non-pharmacological treatment at the time of initial, the number of those who later received such treatment showed an increasing trend over time, which is noteworthy considering the importance of continuous management of insomnia disorder.

## Supporting information

S1 Table Demographic characteristics of patients who received non-pharmacological treatment at first diagnosis, later, or never. (DOCX)
