# Correction: Trends in non-pharmacological treatment for insomnia: A nationwide study

**DOI:** 10.1371/journal.pone.0362107

**Published:** 2026-11-03

**Authors:** Mingee Choi, Junbok Lee, Jeahyung Lee, Suonaa Lee, Eun Lee, Jaeyong Shin

In the Funding statement, the project number for the Ministry of Trade, Industry and Energy (MOTIE, Republic of Korea) under the Technology Innovation Program is listed incorrectly. The correct project number is: Project No. 20018183.
