# Supplementary material for: Trends in non-pharmacological treatment for insomnia: A nationwide study
Source: PLoS One. 2025 Nov 7;20(11):e0334142. doi: 10.1371/journal.pone.0334142 (PMC12594322; doi:10.1371/journal.pone.0334142)
Supplement: S1 Table — (DOCX) [file pone.0334142.s001.docx]

|  | | **Received Non-pharmacological treatment at the first diagnosis** | **Later received Non-pharmacological treatment** | **Without Non-pharmacological treatment** | **P-value** |
| --- | --- | --- | --- | --- | --- |
| **Type of insurance** | **Local subscriber** | 2,951(34.0%) | 7,503(35.4%) | 41,711(33.2%) | <0.001 |
|  | **Employed** | 5,293(60.9%) | 12,379(58.3%) | 78,484(62.4%) |  |
|  | **Medical aid** | 449(5.2%) | 1,336(6.3%) | 5,573(4.4%) |  |
| **Sex** | **Men** | 3,634(41.8%) | 7,192(33.9%) | 49,229(39.1%) | <0.001 |
|  | **Women** | 5,059(58.2%) | 14,026(66.1%) | 76,539(60.9%) |  |
| **Region of residence** | **Metropolitan area** | 4,287(49.3%) | 9,854(46.4%) | 60,857(48.4%) | <0.001 |
|  | **Non-metropolitan area** | 4,406(50.7%) | 11,364(53.6%) | 64,911(51.6%) |  |
| **Income level** | **1(low)** | 1,531(18.3%) | 3,515(17.6%) | 21,211(17.6%) | <0.001 |
|  | **2** | 1,128(13.5%) | 2,928(14.7%) | 17,852(14.8%) |  |
|  | **3** | 1,342(!6.0%) | 3,327(16.7%) | 20,999(17.4%) |  |
|  | **4** | 1,738(20.8%) | 4,230(21.2%) | 26,213(21.7%) |  |
|  | **5(high)** | 2,627(31.4%) | 5,986(30.0%) | 34,530(28.6%) |  |

**S1 Table. Demographic characteristics of patients who received non-pharmacological treatment at first diagnosis, later, or never**
